# Hybrid Hashtags: #YouKnowYoureAKiwiWhen Your Tweet Contains Māori and English

**DOI:** 10.3389/frai.2020.00015

**Published:** 2020-04-09

**Authors:** David Trye, Andreea S. Calude, Felipe Bravo-Marquez, Te Taka Keegan

**Affiliations:** ^1^Department of Computer Science, University of Waikato, Hamilton, New Zealand; ^2^School of General and Applied Linguistics, University of Waikato, Hamilton, New Zealand; ^3^Department of Computer Science, University of Chile & IMFD, Santiago, Chile

**Keywords:** language contact, loanwords, hashtags, hashtag half-life, Māori, New Zealand English, word embeddings, the language of social media

## Abstract

Twitter constitutes a rich resource for investigating language contact phenomena. In this paper, we report findings from the analysis of a large-scale diachronic corpus of over one million tweets, containing loanwords from te reo Māori, the indigenous language spoken in New Zealand, into (primarily, New Zealand) English. Our analysis focuses on hashtags comprising mixed-language resources (which we term *hybrid hashtags*), bringing together descriptive linguistic tools (investigating length, word class, and semantic domains of the hashtags) and quantitative methods (Random Forests and regression analysis). Our work has implications for language change and the study of loanwords (we argue that hybrid hashtags can be linked to loanword entrenchment), and for the study of language on social media (we challenge proposals of hashtags as “words,” and show that hashtags have a dual discourse role: a micro-function within the immediate linguistic context in which they occur and a macro-function within the tweet as a whole).

## 1. Introduction

Languages, like people, rarely exist in complete isolation from one another. One of the most predictable outcomes of language contact, brought about by contact between speakers of (distinct) languages or language varieties, is the adoption of new words from one language (variety) into another. Languages are “leaky” (parallel to Sapir, 2004, p. 29) and speakers act like fluid transmitters of words between the languages they navigate. While linguists have studied loanwords for decades (see work dating back to the 1950s, e.g., Haugen, 1950; Weinrich, 1953), the fruits of this labor can be roughly summarized in three main strands, all of which focus primarily on the borrowing process as a linguistic matter: (1) studies focusing on what is (or can be) borrowed (e.g., Field, 2002; Haspelmath and Tadmor, 2009; Matras, 2009; inter alia), (2) studies attempting to distinguish (if possible) between loanword use and code-switching (e.g., Muysken, 2000; Stammers and Deuchar, 2012; Backus, 2013 and others), and (3) studies which document the adaptation of the loaned material to the internal rules of the receiver language, whether phonological or morphological (e.g., Poplack and Sankoff, 1984; Poplack et al., 1988; Hashimoto, 2019 and references cited within).

In recent decades, a paradigm shift has unfolded in the study of loanwords, which considers linguistic borrowing in its wider sociolinguistic context. In this view, borrowing is not just a linguistic event but also a socially meaningful one, placing both language and speaker at its center. The “socio-pragmatic turn” of loanword study, discussed in a recent *Special Issue* on the topic by Zenner et al. (2019), is shifting to include matters beyond language prestige, such as identity, language ideology, and cultural knowledge (captured by the term “language regard”; see Preston, 2013). Our study seeks to complement this body of work by bringing in the dimension of *language play*. We show that the loanwords in our data are used creatively to signal solidarity with and belonging to an indigenous group, which, despite being previously marginalized, is gaining visibility and status in the wider community. The social dimension of the loanwords we discuss here is undeniably strong and it is virtually impossible to make sense of the borrowing process in this case without recourse to the aforementioned notion of language regard.

The current study examines an unusual language contact situation, as described below. We report findings from an empirically-driven, corpus linguistics analysis of Māori loanwords in (primarily) New Zealand English (NZE) by exploring a purpose-built, large-scale dataset of social media language from the Twitter platform. Examples (1–3)^1^ illustrate the phenomenon in question (loanwords are given in bold text):

(1) Sorry I thought you were **Kiwi** [a New Zealander]. **Aotearoa** is the **Māori** name for NZ [ID 1064121983678406656]

(2) We stand united Native American **Whanau** [family], **kia kaha** [be strong] DakotaAccessPipeline **#haka** [war dance] **#Maori #whanau** #NativeAmerican #united [ID 793003612217577472]

(3) I'm **Pākehā** [European New Zealander] and went to a majority **Māori** primary school. There was lots of incorporation of **#tereo** [the Māori language] and **tikanga** [customs] into everyday activities, set me on path to wanting to live in bicultural **aotearoa** #letssharegood**tereo**stories [ID 959155122289823744]

The language contact situation between the indigenous Austronesian language of te reo Māori and (New Zealand) English presents a unique opportunity to study the flow of words from an endangered, minority-status language (te reo Māori) into a dominant, global *lingua franca* (English). The direction of lexical transfer, especially on the scale of that observed in New Zealand English is, to our knowledge, not comparable to any other language situation previously described (for a detailed description of the nature of the contact situation between Māori and English in New Zealand, see section 3 in Levendis and Calude, 2019 and section 3.1 in Calude et al., 2017).

The study of Māori loanwords in New Zealand English has received intense scrutiny in the literature, especially with regard to newspaper language (Davies and MacLagan, 2006; Macalister, 2006, 2009; Onysko and Calude, 2013), Hansard Parliament debates (Macalister, 2006), children's picture books (Daly, 2007, 2016), TV language (de Bres, 2006), conversation (Kennedy and Yamazaki, 1999), and more recently, online science discourse (Calude et al., 2019b). However, very little is known about the use of Māori loanwords on social media (with the exception of a small sample of tweets in Calude et al., 2019b, and preliminary findings in Trye et al., 2019), which motivates our attention to Twitter data here.

The large body of work cited above has uncovered a number of trends regarding the use of loanwords in New Zealand English. Perhaps the most important one relates to their diachronic use, which strongly suggests that their use is increasing over time (Kennedy and Yamazaki, 1999; Macalister, 2006; Calude et al., 2019a). Moreover, while European settlers initially borrowed flora and fauna words to refer to the new species they encountered upon arriving in New Zealand (e.g., *kiwi, rimu*, and *kauri*), over time, as the new variety of English began to emerge, it started to adopt more material and social culture words (e.g., *marae, tangi*, and *powhiri*; see Macalister, 2006). Secondly, the use of Māori loanwords is driven by Māori women and is largely associated with Māori-related discourse topics (Kennedy and Yamazaki, 1999; de Bres, 2006; Degani, 2010; Calude et al., 2017). Calude et al. (2017) further found that certain loanwords appear to be “more successful” compared to others. Loanword success is defined as the chance of a loanword being used within a receiving language, compared to an existing lexical alternative word native to the receiving language, controlling for the number of opportunities that speakers of the receiving language have to use the concept denoted by the loanword. For instance, loanwords which are shorter than their native English counterpart (in terms of number of syllables, e.g., *pā*/settlement, *tangi*/funeral, *reo*/language) are comparatively more successful, as well as loanwords that encode cultural rather than core meanings (in the sense of Myers-Scotton, 2002). The study also found that linguistic factors interacted with the sociolinguistics ones, such that, for Māori speakers, the ethnicity of the audience had a role to play (when speaking to a Māori-only group, Māori speakers seemed more sensitive to efficiency effects), and, for Pākehā (European) New Zealanders, polysemous loanwords were comparatively less successful than monosemous loanwords (ibid).

In light of what is currently known about Māori loanwords in New Zealand English, we wanted to investigate their use on social media. To this end, we investigated data from Twitter—in part, due to practical considerations (the ease of collecting electronically-searchable data), and in part because this data complements the other types of genres previously investigated. Like spoken, conversational language, Twitter language is (largely) informal, unplanned, non-editable, and immediately available to potential audiences and, like newspaper language, Twitter language is written down. Furthermore, Twitter users span both ends of the formal spectrum, from individuals reflecting their own linguistic style (with regard to lexical content, spelling, word play, etc.) to institutions representing collectives of various sizes (Universities, political parties, etc.) who are perhaps more likely to conform to social norms. However, collecting a corpus of Twitter language for our specific purposes, namely, studying Māori loanwords in New Zealand English, is not without its problems, as discussed in section 3.

One of the most distinctive uses of Māori loanwords in our Twitter corpus, once collected, was the use of *hybrid hashtags*. These are hashtags which involve (at least) one word of Māori and (at least) one word of (NZ) English^2^. Examples include #letssharegoodtereostories (as illustrated in example 3), #kiwigold, #honeyhui, #TreatyofWaitangi, and #beingmaori. We are not aware of any other research that analyses hybrid hashtags specifically, although they are mentioned in passing by Lee and Chau (2018) in their analysis of hashtags on Instagram containing a mixture of English and Cantonese (p. 26). The study of minority languages in social media through hashtag use is not new in itself (see for instance McMonagle et al., 2019), but our focus on combinations of lexical resources from a minority and a majority language in a single hashtag (as opposed to the use of distinct hashtags from different languages in one tweet, as analyzed by Jurgens et al., 2014) has to our knowledge not been studied before. For this reason, the current paper focuses exclusively on the findings uncovered in relation to hybrid hashtags. Before turning our attention to how we built our Twitter corpus and what we found in the data, we first summarize two of the main strands of research questions addressed by recent work on the linguistics of hashtags, in section 2.

## 2. The Linguistics of Hashtags

Linguistic analyses of Twitter and social media discourse are becoming increasingly prevalent as the genre captures the attention of language researchers. One feature which started out on social media, but which is already making its way into other genres (see Caleffi, 2015; Evans, 2015) is the hashtag. Hashtags (denoted with a “#” symbol) have been described as a means of “[categorizing] messages posted on Twitter” (Cunha et al., 2011, p. 58), or of “referring to a topic and creating communities of people interested in that topic” (Caleffi, 2015, p. 67). Adopting a discourse-based approach, Page (2012) conceptualizes Twitter as a “linguistic marketplace,” in which hashtags are a crucial currency. Zappavigna (2011) argues that hashtags function as a “community building linguistic activity” (p. 789) that enables “ambient affiliation” (p. 790).

However, even in this very much emerging body of work, two main preoccupations stand out. First, there are surging debates about the morphological processes which give rise to hashtags. Two main arguments have been proposed so far, which might be succinctly summarized as “hashtags as compounds” (Maity et al., 2016) and “hashtags as hashtagging” (Caleffi, 2015). However, the evidence is still moot with regard to these positions. We return to the word-formation process in section 5.1.

The second open question that has generated interest in the hashtag literature relates to what influences the life-cycle of a hashtag. Given that hashtags are essentially a new brand of “word,” even if only comprising an existing, single word (e.g., #fun), the fact that the word is used together with the “#” symbol and functions as a hashtag distinguishes it both orthographically, semantically and functionally from its use without the “#” symbol. This lexical (re-)birth constitutes a linguistic innovation which means that the hashtag, like all other members of the lexicon of a language, has to “fight for its survival” in order to avoid falling out of use. Romero et al. coin two terms in relation to hashtag life-cycle, namely *persistence*—“the extent to which repeated exposures to a hashtag continue to have a marginal effect” (Romero et al., 2011, p. 695) and *stickiness*—“the probability of adoption based on one or more exposures” (ibid). The term *persistence* is problematic because exposure refers, in practice, to frequency of use of a hashtag, but not necessarily to its likelihood of being seen by other Twitter users (as the word “exposure” suggests), because users do not necessarily read all posts written by users in their Twitter network. *Stickiness* is similarly problematic because of the assumptions encapsulated by the word “exposure.” However, it is certainly possible to use frequency of use of various hashtags on Twitter as a measure of hashtag survival in this genre, assuming that the longer a hashtag is used, the longer its lifespan, life-cycle or survival^3^.

In this paper, we propose (what we believe to be) a more informative measure of a hashtag's success, namely, a hashtag's “half-life,” based on the concept of a word's half-life, introduced by Pagel and Meade (2006). Pagel and Meade define a word's half-life as the amount of time by which a given word has a 50% chance of being replaced by a non-related (non-cognate) form (Pagel and Meade, 2006, 2018; Pagel et al., 2007). By analogy, our notion of a hashtag's half-life refers to the amount of time by which a hashtag reaches half of its total impact (or activity), where “impact” is measured in total number of uses (that is, a frequency of use measure). We return to this in section 4.2.

Regardless of our evaluation of the notions of persistence and stickiness, the most important finding from Romero et al. (2011) in relation to longevity of hashtags pertains to the semantic domain of the various hashtags investigated: hashtags from controversial political topics appear to be more sticky and persistent, whereas hashtags encoding idioms are comparatively less sticky and persistent (2011, p. 701). This finding has informed our own work and we look to the semantic domain of the various hashtags we analyze in relation to hashtag success.

Other studies have also tried to model hashtag longevity by considering various factors. Cunha et al. (2011, pp. 63-64) found an inverse relationship between a hashtag's length and its longevity, and a decrease in longevity associated with the use of underscores in hashtags. Maity et al. (2016, p. 60) investigated two-word compound hashtags (#AB, where A and B are free morphemes) and found that “propagation” of such hashtags is most significantly correlated with an increase in overlap of the lexical content of tweets containing the single-word hashtags (i.e., #A and #B). Tsur and Rappoport (2012) investigate four types of features in relation to hashtag popularity: (1) features concerning the linguistics of the hashtag itself, such as length, position in the tweet, and others, (2) features concerning the content of the tweet containing the hashtag investigated (e.g., tweet length), (3) features to do with the user data of the tweet containing the hashtag in question (e.g., number of followers), and (4) features to do with the temporal patterns of use of the hashtag (normalized weekly counts). They tested these four features as a “bundle” (not separately) and found that, of the four feature types, hashtag content features and tweet content features contributed only a marginal increase in the prediction of hashtag popularity (although they did seem to contribute toward reduced error rates, see p. 649 ff.). The features that do best with regard to predicting hashtag popularity are features to do with user data and timestamps.

## 3. Materials and Methods

This section documents our corpus and the methods we used to build it. We first discuss the Twitter corpus and provide an overview of how we created it, and then focus our attention on the data containing the hybrid hashtags and the sub-corpus we extracted to study these.

### 3.1. Building the Māori Loanword Twitter (MLT) Corpus

The *Māori Loanword Twitter (MLT) Corpus*^4^ was created using a novel technique that relies on a set of query words, instead of following specific users (cf. Keegan et al., 2015) or tracking geolocations (cf. Grieve et al., 2017). This process is briefly summarized below, but a more detailed explanation is given in Trye et al. (2019).

First, we used the Twitter Search API^5^ to obtain 8 million tweets containing one or more query words. The vast majority of these words were compiled by Hay (2018), as part of a study identifying Māori words that most monolingual, English-speaking New Zealanders recognized, even if they did not know their meaning (for the full list of query words, see Table S1). Given the high level of recognition associated with these words, we predicted that they were likely to be used in New Zealand English tweets, and as such, would make a suitable starting point for building the corpus.

However, inspection of the data revealed that many query words frequently occurred in non-New Zealand English contexts, and some were seldom used as loanwords (particularly short, three- or four-letter words with multiple meanings in different languages). We addressed this noise by using supervised machine learning, the problem being analogous to spam classification (see Abayomi-Alli et al., 2019). After manually labeling a sample of tweets for each query word as “relevant” or “irrelevant,” we removed tweets containing query words that were irrelevant more than 90% of the time and trained a classifier to automatically determine when the remaining query words were used in relevant (New Zealand English) contexts. In this way, we could filter out irrelevant tweets to produce a higher-quality corpus.

Drawing on lessons learned from the original study (Trye et al., 2019), some improvements were made to further mitigate noise in the MLT corpus. First, the corpus was enhanced by deploying a Multinomial Naive Bayes model (McCallum and Nigam, 1998) that considered not only unigrams in the feature space (as per the previous study), but bigrams as well. Using the same stratified training set as before, superior Kappa and F-score values were achieved (0.5754 and 0.819, respectively), along with a matching AUC value of 0.872. Additionally, following the removal of tweets classified as irrelevant by the model, 81,830 duplicate tweets were discarded. These duplicates were the result of some tweets containing multiple query words, and being harvested independently by each occurrence.

The final MLT corpus consists of 2,880,211 tweets, comprising 46,827,631 word tokens. In total, these tweets capture linguistic output from 1,226,109 distinct users. A diachronic overview is provided in Table 1.

**Table 1:** Corpus statistics for the MLT corpus, by year.

**Year**	**Tweets**	**Words**	**Users**
2006	8	135	7
2007 △	819	12,872	468
2008 △	5,903	96,665	3,551
2009 △	67,834	1,141,748	38,908
2010 △	142,509	2,310,289	76,713
2011 △	306,389	4,760,881	167,471
2012 △	427,428	6,296,131	241,584
2013 △	446,505	6,630,105	249,388
2014 ▿	345,150	5,254,932	190,181
2015 ▿	315,128	4,847,984	177,482
2016 ▿	240,793	3,741,744	132,867
2017 △	288,779	4,870,311	141,049
2018 △	292,966	6,863,834	143,607
Total	2,880,211	46,827,631	1,226,109

*For the (distinct) Users column, there is some overlap across years, because the same users may be active over multiple years (hence the number of distinct users per year does not match the total in the bottom row)*.

### 3.2. Building the Hybrid Hashtag Sub-corpus

Once collected, we analyzed the MLT corpus for hashtag use. In total, our corpus contains 8,753 distinct hashtags that occur ten times or more (this figure considers alternative spelling, capitalization and punctuation, e.g., macron use, as giving rise to distinct hashtags; therefore, #kiwias and #KiwiAs are counted as separate hashtags).

We manually scanned these hashtags for the presence of Māori and English lexical items, and extracted 287 hashtags that were hybrid. We then discarded hybrid hashtags whose meanings were unclear, even after carefully inspecting the tweets in which they were used (e.g., #kiwifollowspree). Furthermore, we removed hashtags whose meanings were tied to a particular in-group and therefore limited from wider use (e.g., #kiwiPyCon, which refers to a New Zealand-based conference for Python programmers), as well as hashtags denoting specific organizations (e.g., #manaparty), brands (e.g., #maoritv), and sports teams (e.g., #KiwiFerns, used for New Zealand Rugby League).

We primarily wanted to discard hybrid hashtags that were proper nouns because, by and large, these hashtags did not constitute a meaningful linguistic choice (for example, #voteMarama, where “Marama” is the name of a person). However, we did retain six hashtags that were proper nouns, because we wanted to compare their use with content noun phrases and hashtags functioning as other word classes (verbs, clauses, etc.). Of the six proper-noun hashtags, three denote various ethnic or national groups (#MeanMaori, #AotearoaNZ, and #NZMaori), two denote regularly occurring, large-scale, national events (#WaitangiDay^6^ and #MaoriLanguageWeek^7^) and the last hashtag, #TreatyofWaitangi, denotes the most defining event in New Zealand history.

This process whittled down our list of hybrid hashtags from 287 to 135 hashtags. Since the remaining hashtags contained variations in capitalization, macron use, and inflections, we amalgamated them into 81 hybrid hashtag lemmas (e.g., #gokiwis, #goKiwi, and #GOKIWIS were all coded under the single hybrid #gokiwi(s) in our data, and #beingMāori—with a macron—was combined with #beingMaori—without one). The 81 hybrid hashtags were used in 5,684 tweets in total (from the MLT corpus), and posted to Twitter by 3,771 distinct users. These hashtags and their associated tweets comprise the hybrid hashtag dataset—hereafter, the *HH sub-corpus*^8^. For further details about how this corpus was created, please see Supplementary Material, Section 1.

## 4. Results

This section outlines the results of the 81 hybrid hashtags analyzed in the HH sub-corpus. We begin by outlining general linguistic characteristics of the hashtags, specifically the types of loanwords which occur in the hashtags, and the semantic and syntactic function of the hashtags, as well as their lengths. Section 4.2 discusses measures of hashtag success and predictions of hybrid hashtag success in our corpus.

### 4.1. General Linguistic Characteristics of Hybrid Hashtags

The first thing to note about the hybrid hashtags in the HH sub-corpus is that the 81 hashtags are created using only nine Māori loanwords. For the most part, these nine loanwords, given in Table 2, are documented to be among the top ten most frequent loanwords in other corpora of New Zealand English (for example, the *Wellington Corpus of Spoken New Zealand English*, Holmes et al., 1998; the *Matariki Corpus*, Calude et al., 2019a; and the *Māori Language Week Corpus*, Levendis and Calude, 2019). Secondly, they constitute a mix of core and cultural borrowings (following Myers-Scotton, 2002), with a slight skew toward cultural borrowings. Finally, semantically, they tend to denote social culture terms (following the distinctions proposed by Macalister, 2006).

**Table 2:** Linguistic characteristics of the Māori loanwords used in hybrid hashtags.

**Loanword**	**English counterpart(s)**	**Semantic category**	**Core/cultural distinction**
Kiwi(s)	Kiwi fruit, flightless bird or New Zealander(s)	Flora & fauna/social culture	cultural
Māori	(Of) indigenous (origin)	Social culture	cultural
haka	Tribal dance	social culture	cultural
(te) reo	Pertaining to Maori language or to (any) language	Social culture	core
hui	Meeting	Social culture	core
Waitangi	Place name	Proper noun	cultural
Aotearoa	New Zealand	Proper noun	cultural
kai	Food	Material culture	core
kia ora	Hello, thank you, goodbye	Social culture	cultural

*The loanwords are given in order of raw frequency in the HH sub-corpus from most to least frequent. We follow Macalister (2006) for semantic categories of loanwords and Myers-Scotton (2002) for core/cultural distinctions*.

Among the nine loanwords giving rise to the 81 hybrid hashtags extracted, we find that two loanwords, *kiwi(s)* and *Māori*, are significantly more productive in forming hybrid hashtags than all other loanwords. Overall frequency counts and examples are given in Table 3.

**Table 3:** Usage statistics for the nine Māori loanwords present in the set of hybrid hashtags.

**Loanword**	**Raw freq**.	**Hybrid hashtags**	**Total tweets**
kiwi(s)	54	#GoKiwi(s), #proudkiwi(s), #kiwipride, #proudtobe(a)kiwi, #youknowyoure(a)kiwiwhen…	3,487
Māori	12	#beingmaori, #NZMaori, #maorilanguage, #MAORISTYLES, #maoripride…	874
haka	5	#Hakarena, #BanTheHaka, #HakaTime, #thehaka, #lovethehaka	224
(te) reo	3	#LetsShareGoodTeReoStories, #Keep(in)ItReo, #goodtereostories	360
hui	2	#huitweet, #honeyhui	35
Waitangi	2	#WaitangiDay, #TreatyofWaitangi	653
Aotearoa	1	#AotearoaNZ	15
kai	1	#kaitime	15
kia ora	1	#kiaora4that	21
Total	81		5,684

*Loanwords are given in decreasing order of raw frequency in the HH sub-corpus. The hybrid hashtags in the third column are listed according to number of tweets, with the most frequently occurring lemma reported for each one. For the loanwords kiwi(s) and M$\bar{a}$ori there were many more hybrid hashtags than included in the table (only the five most common are shown here; for full details, see Supplementary Material)*.

Many hybrid hashtags contain semantically positive words (e.g. “loyal,” “awesome,” “proud,” “love,” and “good”), which reflect the polarity of the tweet itself. Examples (4) and (5) illustrate this (hybrid hashtags are given in bold text in these and subsequent examples).

(4) @ClaireLHuxley kiwis impress me anyway but that was over and beyond **#proudkiwi** [ID 123993688413188098]

(5) I'm proud to have such a strong heritage, my ancestors were warriors **#maoripride** #proud #Maori #aotearoa #whanau #culture [ID 300417134650068992]

Conversely, there is one hybrid hashtag, #BanTheHaka, which is (nearly always) explicitly negative. The haka is a Māori tribal dance that is routinely performed (among other occasions) before international rugby matches, and it is in this capacity that it has gained considerable attention on the world stage. However, the practice has attracted controversy from people who see the behavior as unnecessarily aggressive or intimidating. Example (6) provides an opinion to this effect and example (7) links the haka to an “unfair advantage” to the team performing it. Both these tweets align themselves with the literal and most likely, the original meaning captured by the hashtag #BanTheHaka, which is to express a negative attitude toward the haka.

(6) The Haka has never been “Respectful”! It's always been aggressive! **#BANTHEHAKA** [ID 796629023887622144]

(7) @gwladrugby. The Haka is an unfair advantage for NZ to be able to perform b4 the game, should be able to respond how u wish ! **#banthehaka** [ID 128792760386985985]

However, another tweeter in our corpus uses the hashtag to join the discussion surrounding the practice of the haka, but with the aim of presenting the opposite view; namely, writing in support of the tradition.

(8) #BanTheIgnorance instead of ban the haka. Do some research next time you insult an entire culture **#BanTheHaka** [ID 665815361694994432]

These examples illustrate two facets of hashtags. First, hashtags need to be interpreted by examining the global (macro) context within which they are used (here within the entire tweet, not just with reference to the phrase or clause they are part of). Secondly, they can have a dual function within this context of use, one of these functions being the semantic expression of a particular meaning, for instance, in examples (6) and (7), the expression of a negative attitude toward the performance of the haka, and a second function being a discourse affiliative role, namely of contributing to an existing discussion or community of practice (as also argued by Cunha et al., 2011; Caleffi, 2015). Our examples show that the two functions can co-occur without conflict in many tweets [examples (6–7) are such cases], but that it is also possible for the two functions to appear in conflict with each other [as in example (8)], when the literal meaning expressed by the hashtag violates the propositional content of the tweet. In such cases, the conflict is resolved by having the discourse affiliative function override the semantic expression of the hashtag (rendering the hashtag's semantic content moot). We return to these points in the Discussion section.

Given the findings discussed by previous literature on hashtags more generally (see section 2), we also investigated four linguistic properties of our set of hybrid hashtags, including hashtag length and semantic domain (as per previous studies). In addition, we considered whether the hashtags had multiple distinct variables (before amalgamating the lemmas), and looked at each hashtag's syntactic word class^9^.

**Figure 1:**
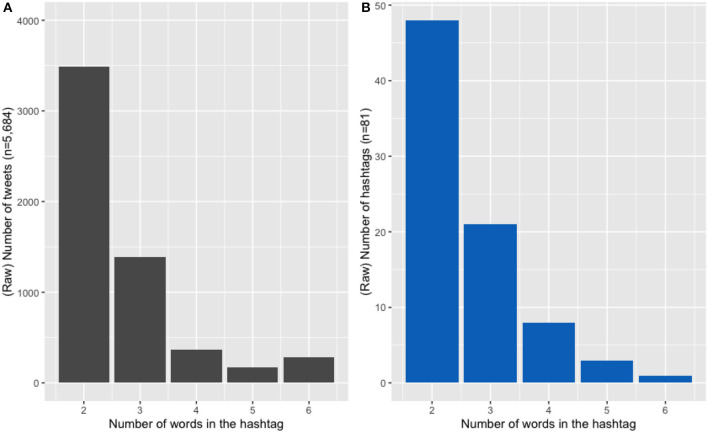
**(A)** Distribution of hashtag length across number of tweets. **(B)** Distribution of hashtag length by (hashtag) type.

The first linguistic characteristic coded was hashtag length, in number of words (following other work analysing hashtag length, namely Cunha et al., 2011; Tsur and Rappoport, 2012; Maity et al., 2016). Figure 1^10^ Illustrates the distribution of lengths in the HH sub-corpus (by both number of tweets, Figure 1A, and by distinct number of hashtags, Figure 1B). As can be seen, these lengths range between two and six words, with most hybrid hashtags consisting of two words.

Next, as discussed in section 3.2, some hashtags had multiple variants (due to slight differences in capitalization, macron use, and/or inflections), whereas others consisted of only one form. For example, the hashtag #flyingkiwis has three variants, which vary in their use of capitals and singular/plural forms: #FlyingKiwis, #flyingkiwis, and #flyingkiwi. As noted above, we did not want to count these hashtags as being distinct so we merged them into the same hashtag lemma. Our corpus of 81 hashtags contains slightly more hashtags with unique forms (*n* = 46) than with multiple variants (*n* = 35). However, the hashtags with multiple variants appear to be used in a higher number of tweets overall (see Figure S1).

**Figure 2:**
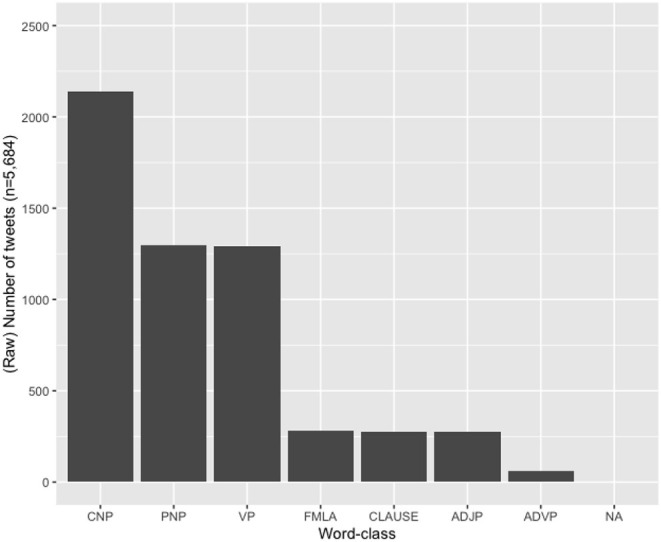
Distribution across various word-classes in the hybrid hashtag set (CNP, common NP; PNP, proper NP; VP, verb phrase; FMLA, formulaic phrase; CLAUSE, full clause; ADJP, adjective phrase; ADVP, adverb phrase; NA, unsure).

Third, we consider word-class possibilities for the hybrid hashtags. Table 4 details the various word-class possibilities realized in our data and provides examples to illustrate these. Figure 2 shows a frequency distribution of these possibilities in the HH sub-corpus (in terms of number of tweets).

**Table 4:** Word-classes of the various hybrid hashtags in the HH sub-corpus.

**Word-class**	**Hashtag example**	**Example of tweet containing hashtag**	**Num hashtags**
Adjective Phrase (ADJP)	#kiwiproud	See you tonight Sydney City! Look for the wasted guy doing the haka. #KiwiProud hahahaha. [ID 523052566855184384]	3
Adverb Phrase (ADVP)	#kiwias	Usual weekend of sports entertainment resumes in NZ on @skysportnz this wkend! #SuperRugby #NRL #NBL #ALeague #kiwias #kiwi #kiwiana #sport! [ID 441819484534210560]	2
Common Noun Phrase (CNP)	#thehaka	So I don't know anything about #Rugby but I do know #TheHaka; Kiwi yr7 teacher had us do it :D Manly rugby boys doing it's a better view tho [ID 658053257416318976]	43
Formulaic Phrase (FMLA)	#kiaora4that	@tttrips Yeah…nah,not enuff gas bro but #kiaora4that anyway. He whakaaro Rangatira tena. [ID 272442027508117505]	5
Full Clause (CLAUSE)	#kiwiscanfly	Good luck to the kiwi triathletes racing in the European junior cup at Eton Dorney tomorrow @ETUtriathlon @TriathlonNZ #kiwiscanfly! #NZ [ID 373565680093630465]	6
Proper Noun Phrase (PNP)	#NZMaori	Going off to see the #nzmaori game today. Probability be more expat kiwis at the game than locals. [ID 396997290541326336]	6
Verb Phrase (VP)	#maorifyNZ	In order to #Maorifynz I will be swapping out my own Pakeha DNA with some spare M$\bar{\text{a}}$ori genes that Miriama Kamo has. [ID 90561843961147392]	13
N/A			3
Total			81

**Figure 3:**
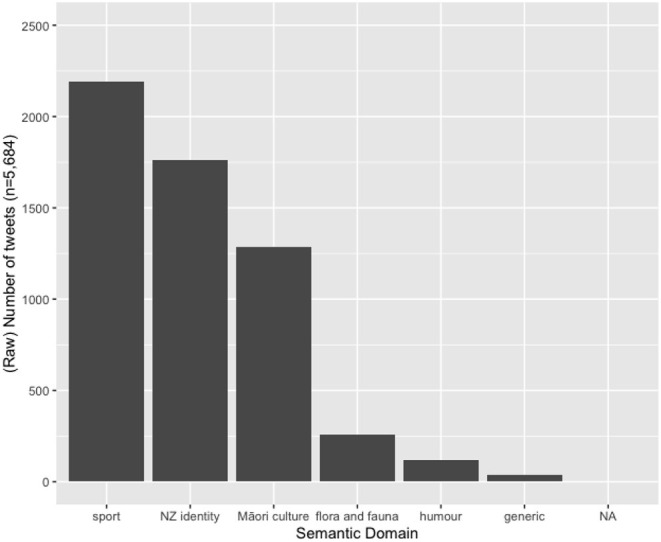
Distribution across various semantic domains in the hybrid hashtag set.

Finally, we turn to the semantic domain of our hybrid hashtags. In accordance with claims by Macalister (2006) for other genres of New Zealand English, we also find that the hybrid hashtags are used to reference New Zealand identity, (NZ) flora and fauna and humor (see also Macalister, 2002), but in addition, we find that they are commonly used in sporting contexts. Table 5 exemplifies each of the semantic domains uncovered in the HH sub-corpus, and Figure 3 gives their frequency distribution.

**Table 5:** Semantic domain of the various hybrid hashtags in the HH sub-corpus.

**Semantic domain**	**Hashtag example**	**Example of tweet containing hashtag**	**Num hashtags**
Flora and Fauna	#kiwiberries	I just discovered #kiwiberries, they are exactly what they sound like a small bite sized kiwi with no fuzz, best things ever! [ID 121230747351781377]	7
Generic	#kaitime	Honestly, no one can tell I'm Maori until they see me when there's seafood up for grabs… until then I'm pretty much plastic #kaitime [ID 91506535969021952]	2
Humor	#replacemovie quoteswithkiwi	my kiwi brings all the boys to the yard… #replacesongwordswithkiwi [ID 106461006527602689]	6
Māri culture	#keepinitreo	next week all orders at the drive thru in te reo maori #keepinitreo [ID 226445367913365504]	17
NZ Identity	#kiwislang	Caught myself saying something with a slight English accent today…I need to hear some kiwis ASAP #kiwisinlondon #kiwislang [ID 552521136127639554]	28
Sport	#kiwigold	@andreahewittnz does it again with a convincing first place at #ITU #GoldCoast #GoldCoastTri #kiwi #kiwigold [ID 850741519753596928]	20
N/A			1
Total			81

This was by far the hardest linguistic factor to code in our data. Two main sets of problems made the coding difficult. First, some hashtags seemed to belong to multiple semantic categories, either because different tweeters used the hashtag in different ways, or because the same tweeters varied their use of the hashtag (or sometimes a combination of both), as shown in examples (9–11). Secondly, the meaning of the hashtag was not always transparent, nor was its use in the tweet. In all cases, we chose the domain that appeared to be the most dominant in the HH sub-corpus (i.e., the domain that applied to the most tweets containing that particular hashtag).

For example, consider the hashtag #kiwiquestion. This hashtag was mostly used by the same tweeter, but sometimes in reference to (native) flora and fauna (9) and sometimes denoting NZ identity (10):

(9) Here we go, our **#KiwiQuestion** of the day: What are thought to be the kiwi bird's two closest relatives? [ID 288571983359262720]

(10) **#KiwiQuestion** What do the stars on the New Zealand flag represent? Answer for a #free Shisha from Kiwi. Smokers unite! #Maadi #freestuff [ID 293282293051703297]

Example (11) shows the use of the same hashtag by a different tweeter, in a completely different context (to ask a question about eating kiwifruit, which falls under the “flora and fauna” category):

(11) Random I know but do you leave the skin on a kiwi fruit when eating it or peel it off? **#kiwiquestion** [ID 177022614559141888]

However, we classified this hashtag as “NZ identity” because most of the tweets were similar to example (10).

In order to alleviate the problems we had in assigning a (single) semantic domain to each hybrid hashtag, we verified our choices by training word embeddings on the MLT corpus and visualizing the semantic neighborhood of the hybrid hashtags in question.

Word embedding algorithms utilize principles of distributional semantics—the notion that similar words occur in similar contexts—to model relationships between words. These algorithms have gained prominence in the field of Natural Language Processing (NLP) in recent years, and are widely regarded as a useful tool for linguistic analysis (when used appropriately). However, word embeddings are not without their limitations, as discussed by Bowern (2019) (among others). In particular, the results are brittle, require large corpora and do not support word sense disambiguation (which has repercussions for polysemous loanwords such as *kiwi*). In the context of studying language change, Bowern (2019) argues that word embeddings obscure critical data, overlooking the variation that is the input to change. We use word embedding plots for a different purpose here, namely, to help us glean the dominant semantic domain within which a hashtag occurs (given that we already know of its polysemy, following qualitative analyses of the data).

We trained word embeddings on the MLT corpus and identified the closest words in the semantic space to each of our hybrid hashtags. It was important to train embeddings on the MLT corpus rather than the HH sub-corpus because word embeddings work best with a large amount of training data. We implemented the *Word2Vec* algorithm (Mikolov et al., 2013) using Python's *Gensim* library (Rehurek and Sojka, 2010). After fine-tuning hyper-parameters, a CBOW architecture with negative sampling was chosen (*n* = 5), together with a window size of 15 and dimensionality of 200. This window size was chosen by maximizing the Mean Reciprocal Rank (MRR) of a list of chosen word-pairs (48 near-synonymous Māori/English word-pairs). The embeddings were then projected into two-dimensional space, using t-SNE (t-Distributed Stochastic Neighbor Embedding), a machine learning algorithm that preserves the distance between vectors when their dimensionality is reduced (see Maaten and Hinton, 2008).

**Figure 4:**
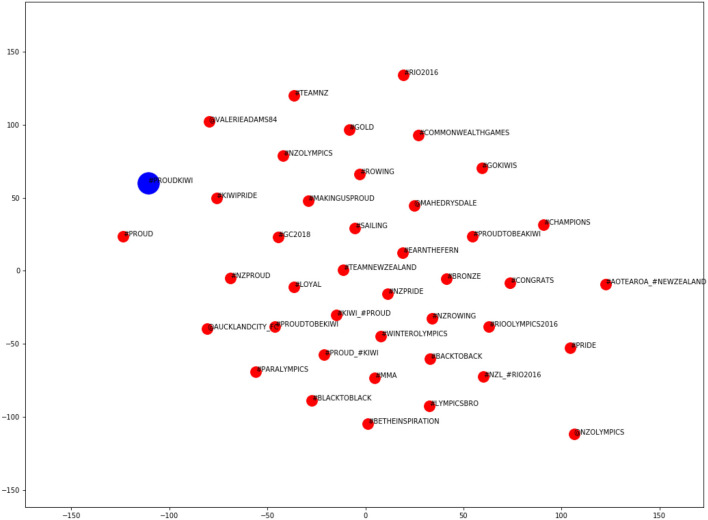
Word embedding plot for the hashtag #proudkiwi.

**Figure 5:**
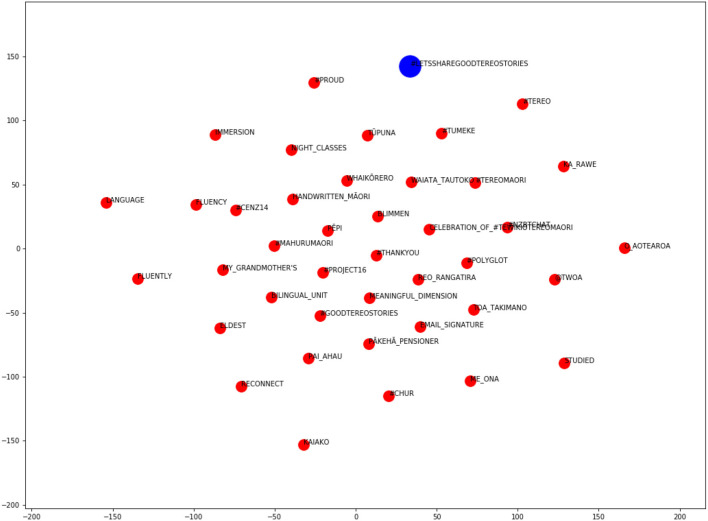
Word embedding plot for the hashtag #letssharegoodtereostories.

In the resulting plots, the blue dot represents the target hybrid hashtag and the red dots represent the 40 closest words in the semantic space (those with the highest cosine similarity), which may consist of (native) English and/or Māori words. Figures 4, 5 show how these plots can help to identify the hashtag's semantic domain.

It is clear from Figure 4 that the hashtag #proudkiwi pertains to sport. The semantic neighborhood includes the names of several famous New Zealand athletes (e.g., Mahe Drysdale, Andreea Hewitt, Lisa Carrington, George Bennett), specific sporting competitions (e.g., #London2012 Olympics), different sports in which New Zealanders excel (e.g., cycling, sailing, golf, rowing), references to "NZparalympics" and related hashtags (e.g., #EarnTheFern, #Gold).

Figure 5 relates to the hashtag #letssharegoodtereostories, and shows a number of Māori cultural terms, such as *#tereo* (the (Māori) language), *tupuna* (ancestors), *kaiako* (teacher) and *whaikōrero* (formal speech). Other words in the neighborhood are related to learning and promoting the Māori language (e.g., “immersion,” “fluency,” “bilingual_unit,” “reconnect,” “meaningful_dimensions,” and “night_classes”), and/or to people's attitudes (e.g., “proud,” *tu meke*/“too much”). From inspecting the plot, we can glean that this hashtag relates to the “Māori culture” semantic domain.

### 4.2. Measuring Hashtag Survival/Life-Span

Given that the HH sub-corpus spans a period of 10 years, it is possible to investigate diachronic trends in the use of the hybrid hashtags extracted. Some of the hashtags rise more rapidly (e.g., #growingupkiwi, #youknowyoureakiwiwhen) or less rapidly (e.g., #kiwipride, #MāoriLanguageWeek), reach a peak and then decrease into disuse. Other hashtags have a cyclic life-span, whereby they are only used in specific months of the year recurrently, and not in other months (e.g., #TreatyofWaitangi). In general, as also noted by Maity et al. (2016), hashtags are highly transient and their life-span tends to be short. The hybrid hashtags in the HH sub-corpus are no exception to this trend.

We calculated Kendall Tau tests to check the status of the 81 hybrid hashtags in our set (by considering the more accurate counts of frequency per month), and found that 18 were statistically significantly increasing in use (#WaitangiDay, #proudkiwis, #letsshregoodtereostories, #kiwifruit, #hakarena, #kiwiproud, #kiwilove, #kiwias, #kiwisongs, #maorilanguage, #hakatime, #thehaka, #maoripride, #meanmaori, #kiaora4that, #proudmaori, #newkiwiburgersong, and #kiwiberries). The Kendall Tau test results for all 81 hashtags are reported in Table S2.

**Figure 6:**
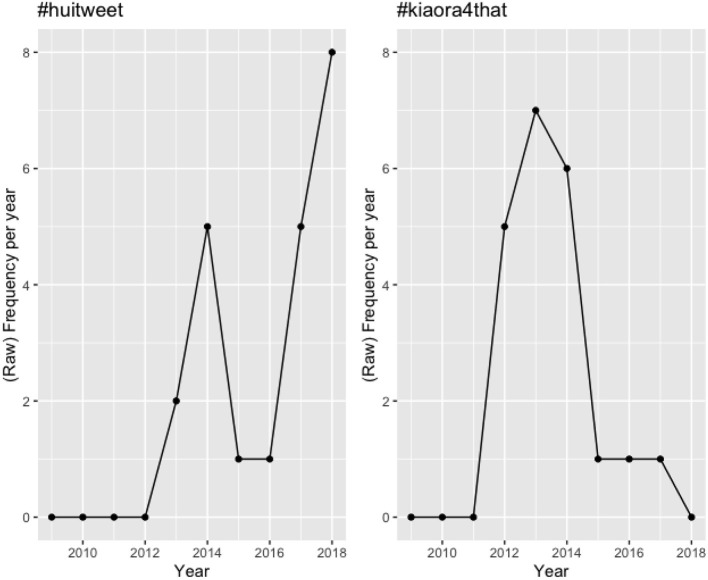
Diachronic trend for #huitweet and #kiaora4that in the HH sub-corpus.

Studies which investigate hashtag survival use raw frequency of occurrence as a measure of the popularity of a given hashtag (e.g., Cunha et al., 2011; Tsur and Rappoport, 2012; Maity et al., 2016). There are few attempts to check these frequencies of use as they unfold over time—Maity et al. (2016) is a notable exception. In their work, Maity et al. (2016) track hashtag use by recording the (raw) number of occurrences of hashtags across weeks. However, one problem with this raw measure is that it does not distinguish between hashtags that occur across the same total number of weeks but which have a very different frequency distribution across those weeks. See, for example, the diachronic plots for the hybrid hashtags #huitweet and #kiaora4that in Figure 6^11^.

Both these hashtags have a life-span of 5 (years), yet their use is very different within the 5-year period in which they occur. We propose an alternative measure of hashtag life-span (or survival) which takes into consideration both the duration that the hashtag is used for, as well as its relative activity or impact (i.e., how much it is used) in that period. Our notion of a hashtag's half-life is based on the idea of a word's half-life proposed by Pagel and Meade, which captures the point by which a given word-form has a 50% chance of being replaced by a non-cognate form (Pagel and Meade, 2006, 2018; Pagel et al., 2007). Analogously, the half-life of a hashtag captures the duration by which a given hashtag achieves 50% of its impact or activity (measured in frequency of use).

**Figure 7:**
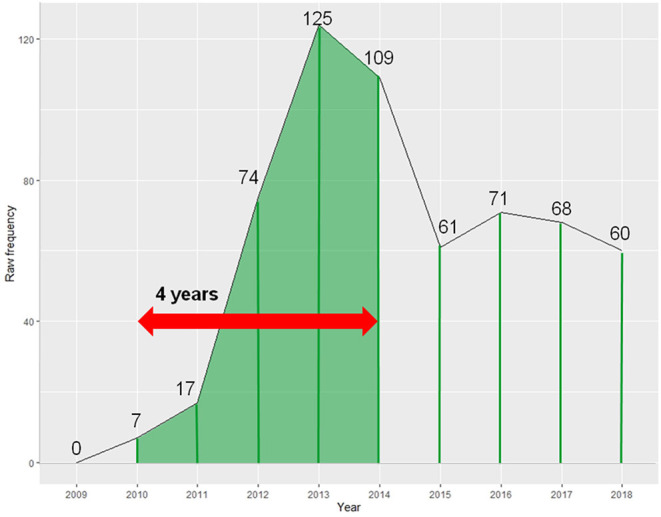
Calculating the half-life of a hashtag.

In practice, this measure can be operationalized separately for each hashtag, by calculating the amount of time it takes for a given hashtag to reach the half-point of the probability density function of its total observed frequency (during the period investigated). We did this in our data by using formulae in an Excel spreadsheet. The process is illustrated graphically in Figure 7, and mathematically, as follows. The hashtag in Figure 7 has been simplified to show half-life in years (of which there are 10) for illustrative purposes—but we do not use years as our preferred time measure (we return to this further below). For now, let's consider the general process of calculating the half-life measure. The hashtag in Figure 7 has a total frequency of use of 592 (occurrences), so it reaches its half-life at 592/2 = 296 uses. The half-life measure is a temporal stamp, so we need to calculate the time it takes (starting from its very first use in the corpus in 2010) for the hashtag to reach the frequency of 296 occurrences (in 2014), which turns out to be 4 years (because 7_2010_ + 17_2011_ + 74_2012_ + 125_2013_ + 109_2014_ > 296).

Returning to Figure 6, #huitweet has a half-life of 4 years, whereas #kiaora4that has a half-life of 1 year, reflecting the different nature of their frequency distributions. We chose to measure half-lives of hybrid hashtags in our corpus across number of months in a bid to obtain the most fine-grained measurement (more accurate than years) while still avoiding data sparsity issues (which arose when considering number of weeks).

It is important to note that both existing measures of hashtag survival and the new measure we propose here (hashtag half-life) suffer from the drawback that they do not accurately capture the life-cycle of recently-coined hashtags. Current measures cannot say anything meaningful about the survival of such hashtags, given that we may not have seen their peak, or have been able to learn anything about the course of their use in the little time that they have existed on Twitter.

In our dataset, the half-life (estimated in number of months) values range between 0 months (for 13 distinct hashtags) and 79 months (for #kiwisdofly). See Figure S2 for a frequency distribution of the various half-lives calculated for each of our 81 hybrid hashtags.

**Figure 8:**
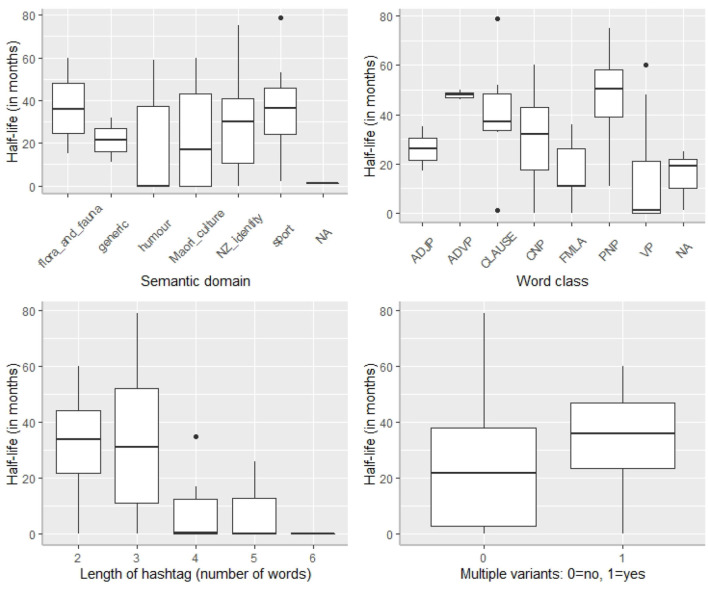
Frequency distribution of half-lives of our 81 hybrid hashtags.

One obvious question to ask is whether there is any relationship between the various linguistic characteristics of the hashtags analyzed in the HH sub-corpus and their respective half-lives. Figure 8 provides box-plot summaries of the various half-lives across each of these characteristics (semantic domain, word class, length of hashtag, and multiple variants).

The plots indicate that there are differences between the various types of hashtags (with respect to length, word-class, semantic domain, and whether or not hashtags are expressed by unique forms) and their respective half-lives. Since it is possible that all of these factors may influence a given hashtag's half-life (and, most likely, many other factors not coded here do too), we first used a Random Forest analysis implemented by the Boruta package in R (Kursa and Rudnicki, 2010) to check which factors are significantly associated with half-life scores. Boruta is a Random Forest technique which samples with replacement (unlike Conditional Inference Trees, see Baayen, 2008; Levshina, 2015).

**Figure 9:**
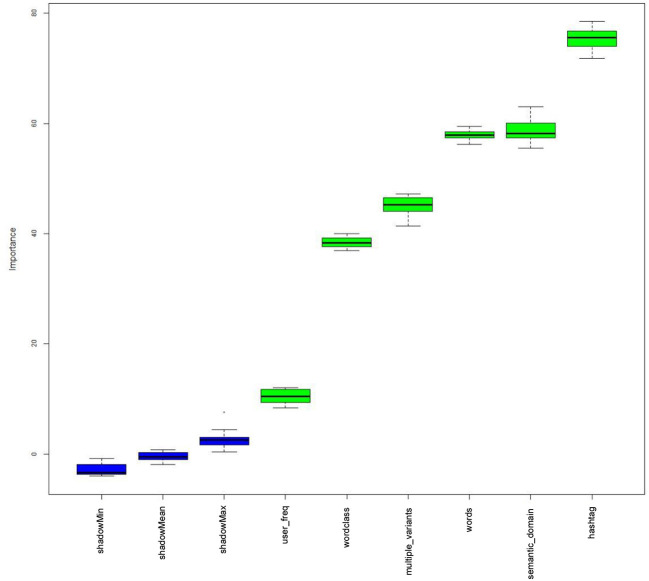
Boruta plot showing the factors which are deemed to be relevant to half-life scores.

Before running the Boruta function, we collapsed our word-class variable into two categories, namely, *nominal* (common and proper noun phrases) and *non-nominal* (all other classes: verb phrases, adverb phrases, adjective phrases, clauses, and formulaic hashtags). We also collapsed the semantic domain variable into four categories, namely, NZ identity, Māori culture, sport, and *other* (which includes humor, flora and fauna, and generic). This updated categorization system was adopted in order to ameliorate the under-representation problems of the original categories (for example, there were only two adjective-phrase hashtags). In addition to our four linguistic characteristics, we also included the hashtag, the user and the user frequency for each hashtag. This is because the same user is sometimes associated with multiple (distinct) hashtags, and different users will tweet the various hashtags with different frequencies. Figure 9 gives the resulting plot. A description of each of these variables is given in Table S3.

We then built a step-up Generalized Mixed-Effects Model with a Quasi-Poisson distribution^12^, modeling the half-life values obtained using the predictors that were deemed significant in the Boruta analysis (all except “user”). We thus included hashtag as a random variable, and the following remaining variables as fixed effects: semantic domain, length of hashtag, word class of hashtag, whether or not the hashtag had a unique form or multiple variants, and user frequency. The final minimal adequate model contained three factors: semantic domain, length of hashtag, and word class of hashtag, and a three-way interaction between these (see Figure S4, for further details). We inspected Cook's Distances and did not find outliers (see Figure S4). Table 6 provides a detailed summary of the model. In general, increased hashtag length and non-nominal word-class are both associated with lower half-life scores; however, this effect is mediated by semantic domain of the hashtag. Non-nominal hashtags denoting sport or other concepts tend to have shorter half-lives compared to non-nominal hashtags denoting NZ identity. Conversely, nominal hashtags show the opposite trend: those denoting NZ identity have longer half-lives compared to those denoting sport or other concepts. Three-way interactions are notoriously difficult to interpret and these findings are only preliminary; more data are needed to confirm the trends.

**Table 6:** Detailed summary of the GLMM model.

**Predictor**	**Value**	**SE**	**DF**	***t*-value**	***p*-value**
(Intercept)	2.811575	0.288647	4096	9.740528	0
words	0.066247	0.073783	62	0.897869	0.3727
wordclass_nonnominal	−0.2031	0.896049	62	−0.22666	0.8214
**semantic_domain_** **New_Zealand_identity**	**−9.48639**	**5.155466**	**62**	**−1.84006**	**0.0705**
**semantic_domain_other**	**23.6385**	**4.089416**	**62**	**5.780409**	**0**
semantic_domain_sport	0.130252	0.333948	62	0.390037	0.6978
words: wordclass_ nonnominal	−1.08378	0.616854	62	−1.75695	0.0839
words: 3 semantic_domain_ New_Zealand_identity	0.702501	2.574691	62	0.272849	0.7859
**words:** **semantic_domain_other**	**−12.5044**	**2.037805**	**62**	**−6.13622**	**0**
**words:** **semantic_domain_sport**	**−0.3489**	**0.11128**	**62**	**−3.1353**	**0.0026**
**wordclass_nonnominal:** **semantic_domain_** **New_Zealand_identity**	**15.62723**	**5.242248**	**62**	**2.981016**	**0.0041**
**wordclass_nonnominal:** **semantic_domain_other**	**−29.0146**	**4.253724**	**62**	**−6.82098**	**0**
**wordclass_nonnominal:** **semantic_domain_sport**	**2.066326**	**0.915875**	**62**	**2.256121**	**0.0276**
words: wordclass_nonnominal: semantic_domain_ New_Zealand_identity	−3.87852	2.658266	62	−1.45904	0.1496
**words:** **wordclass_nonnominal:** **semantic_domain_other**	**13.65776**	**2.19546**	**62**	**6.220909**	**0**
**words:** **wordclass_nonnominal:** **semantic_domain_sport**	**3.046891**	**0.623952**	**62**	**4.883211**	**0**

*Significant predictors are emphasized in bold*.

It is important to emphasize that the models were not built for testing predictive power, but to test the influence of the variables. Given a particular hashtag, we would not expect the model to accurately predict its half-life; rather, the hypothesis tested here is whether or not a certain linguistic characteristic is statistically more likely to be associated with a higher half-life. Furthermore, due to practical constraints, the model lacks sociolinguistic predictors related to the users (such as gender, ethnicity, and status), which are also likely to influence hashtag life.

## 5. Discussion

The previous section details our findings in relation to the set of hybrid hashtags found in the MLT corpus over the 10-year period investigated. While we cannot make any claims regarding the exhaustiveness of the Māori-English hybrid hashtags used on Twitter in general—our set of hybrid hashtags pertains only to the tweets obtained by means of the set of query words used to search the Twitter API—we believe that the data analyzed here can inform wider discussions of hashtags (beyond hybrid hashtags themselves) and current understanding of loanwords (as a linguistic and social phenomenon). We focus the discussion on three main issues.

### 5.1. Word-Formation in Hybrid Hashtags

As mentioned in section 2, there is divided opinion in the literature regarding the morphological word-formation process which gives rise to hashtags (see especially Caleffi, 2015; Maity et al., 2016). The most intuitive way to classify the formation of hashtags is by recourse to compounding, which is a problematic process in itself (see discussion in Bauer, 2017), but which appears to be among the most productive mechanism for creating new words in English. Certainly, some examples of hashtags in our data fit the compounding strategy well; see (12) and (13).

(12) I love a good Kiwi accent. test = tist six = sex **#kiwiaccent** [ID 58156310386065408]

(13) I remember going to the Zoo growing up and rarely seeing the Kiwis. Awesome news for the species! **#kiwibird** #kiwisandiegozoo… [ID 526886414118842369]

In (12), the common noun *Kiwi accent* parallels an existing productive compounding schema, e.g., British accent, Australian accent, American accent, as does the noun *kiwi bird* in (13), e.g., blackbird, bluebird, bellbird, tropicbird, secretarybird. These compounds are right-headed, as is typical of English compounds, and comprise a noun-noun combination, also a highly utilized combination in English. The feature which makes these compounds distinctive is the combination of lexemes from distinct languages, Māori and English—but this type of combination has been documented as a productive word-formation strategy in other genres of New Zealand English (see Degani and Onysko, 2010).

However, compounding cannot account for hybrid hashtags that function as phrasal units exhibiting a productive syntactic frame, as evidenced by the variations we see in the hashtags' form [sometimes including the determiner, as in (15) and sometimes without it, as in (14)], but also by the existence of close alternative hashtags, such as (16) and (17). The lack of internal consistency violates one of the criteria proposed by Haspelmath (2011, p. 7) for word-hood. A second principle which appears to be potentially violated is that of potential pauses. Words are typically not able to include pauses (Haspelmath, 2011, p. 6). Of course, this is difficult to check in Twitter—a written language medium—but hashtags like #kiwiasbro, when uttered aloud are understood as separate words by speakers (*kiwi, as, bro*). This leads us to question the status of hashtags as words in the first place.

(14) So happy of our wee country! Best Olympics & now another gold, well done nz! So proud to be a kiwi #2012Olympics **#proudtobekiwi** #nzolympics [ID 234994140339900416]

(15) Double Gold! No voice and one bloody proud kiwi! #GoKiwi @nzolympics **#proudtobeakiwi** [ID 231354255653621760]

(16) #kiakaha today @RealStevenAdams in your first #NBA start. Play hard, enjoy the game. **#kiwiproud** [ID 400777324062187521]

(17) **#ProudKiwi** im a proud kiwi rt if you are to favorite if you from auckland [ID 235017500000133121]

Even more problematic hashtags are those which span entire clauses, as in (18) and (19). The complex internal structure of clausal hashtags is also noted by Caleffi (2015) and forms the main evidence for her proposal that hashtags represent a completely distinct word-formation process, which she terms *hashtagging*.

(18) **#kiwisareawesomepeople** for protecting their native animals like kiwis,kea,kekapo,weka,morepork [ID 25866163769]

(19) Its kinda depressing that I might be allergic to Kiwi. **#ilovekiwi** [ID 474333666814877696]

The meanings of hashtags in the examples above can only be decoded by taking into consideration the meaning and syntactic role of the individual words comprising the hashtag, in the same manner as any other clause in English. The only difference is the orthographic appearance of the hashtag, which uses the “#” symbol and lacks spaces between words. Moreover, the syntactic structure of the hashtag can be expanded to richer and more elaborate hashtags, e.g., #ilovefunnykiwis or #heloveskiwis, to create novel hashtags, in a highly productive fashion, reminiscent of typical English phrasal structures.

We question the status of hashtags as words and suggest that hashtags are, at best, artificial words, and therefore outside the scope of the usual morphological formation processes that would typically underpin the formation of (legitimately) new words in a language system^13^. Given their function in discourse, these units must “look,” orthographically, like individual words (by having spaces removed between their components) in order to facilitate searchability and discovery by other online community members. However, linguistically, we argue that they should not be analyzed as actual words because they are derived from a number of distinct processes (some of which are indeed akin to compounding, while others are not), and interpreted by recourse to analysis of the individual components within each use.

### 5.2. Function of Hybrid Hashtags in Discourse

Previous work on loanwords identifies a number of linguistic and non-linguistic reasons for the adoption of lexical material from one language into another. These include filling lexical gaps in the receiver language or lexical gaps of bilingual speakers, economy of expression, expression of identity, language regard, and so on (Poplack, 2018, chapter 11 and others).

One factor which has been relatively under-represented in the literature on loanwords (but see Macalister, 2002 for a handful of examples from New Zealand English) is the dimension of humor and language play. Language play and creative uses of linguistic resources (see Zirker and Winter-Froemel, 2015 and papers cited within) have been documented in monolingual contexts of word formation (Renner, 2015) and in English-German bilingual puns (Stefanowitsch, 2002; Knospe, 2015), but to our knowledge, they are largely absent from studies of loanwords. Given the link between creativity and bilingualism (see overview in Kharkhurin, 2015), it is perhaps not surprising that loanwords illustrate creative language use and language play.

We found that Twitter is a particularly rich genre for investigating language play in loanword use. Although we devised a specific semantic function category to include hybrid hashtags whose primary function is that of invoking humor, many of the other uses of hybrid hashtags appeared to also exhibit language play and humorous undertones, even if this was not their primary function. As an illustration of this phenomenon, consider example (20).

(20) it's time to start focusing on regional economic development for our whanau and runanga says @ngaitahu **#honeyhui** [ID 760990045389987840]

In (20), the Māori word *hui* is roughly translated in English as “meeting” or “gathering.” The hybrid hashtag #honeyhui is used in the above tweet by MBIE (*The Ministry of Business, Innovation and Employment*) as a creative reference to the English concept of a “working bee,” bringing a light-hearted touch to an otherwise serious and controversial effort to improve the economic situation of regional councils and (New Zealand) families. The councils and families in question are referenced by means of Māori loanwords (the word *whānau* refers to family and extended family members, and the word *runanga* refers to a council). The use of Māori loanwords for these concepts is socially meaningful because it invokes an inclusive practice, emphasizing the fact that the effort aims to improve the economic development of all regional councils and families; the use of Māori loanwords references those councils and families predominantly made up of Māori (and thereby explicitly referencing groups which might have previously been marginalized from such an effort). The discourse function of the hybrid hashtag #honeyhui has less to do with categorizing the tweet or with signaling group affiliation, and more to do with bringing together two distinct worldviews and points of reference, in a suggested unified action to improve economic development. The hashtag functions as a softening device (achieved through light-hearted humor), aimed at defusing tension in a delicate and socially-charged situation. Other phenomena unique to computer-mediated communication, such as emojis, can play a similar role in the diffusion of tension (for further discussion, see Evans, 2017). The example shows the richness of meaning that can be derived from loanword use and the different layers of interpretation arising from this use.

Additional examples of hashtags with humorous undertones can be seen in the use of the hashtags #youknowyoure(a)kiwiwhen and #growingupkiwi, in examples (21) and (22), respectively. Both these tags primarily discuss issues of New Zealand identity (and are categorized as such in our analysis), but they also bring in a playful dimension. In (21), the user laments the Marmite shortage that occurred when Sanitarium ceased production of Marmite, due to factory damage caused by the 2011 Christchurch earthquake. This shortage caused an uproar in the New Zealand community because the New Zealand brand of Marmite is seen an icon of kiwi culture. The hashtag #youknowyoure(a)kiwiwhen facilitates the user's attempt to poke fun at the problem of grieving the loss of marmite by implying that only a New Zealander would understand this loss and by hinting (implicitly) that the magnitude or validity of this loss is underestimated by those who are not New Zealanders.

(21) **#youknowyourekiwiwhen** you grieve the loss of marmite [ID 427393399855923200]

(22) **#growingupkiwi** being a skinny white kid in a Primary school Kapa Haka group [ID 621264554266243072]

In (22), #growingupkiwi is similarly used to focus attention on the experience of being a New Zealander, and presents this experience as distinct and perhaps misunderstood by outsiders. *Kapa haka* groups are traditional Māori performance groups, typically made up of Māori children, but in recent years, children of European descent have started to join in too (referenced by the comment about being the “skinny white kid” among the predominantly dark-skinned Māori children in the group).

Unlike #honeyhui, the hashtags #youknowyoure(a)kiwiwhen and #growingupkiwi are humorous not because of word-play, but because they describe relatable, shared experiences of being a New Zealander and being raised in New Zealand.

The examination of Twitter data may be more conducive to discovering creative uses of loanwords compared to other genres because of the informal and potentially anonymous^14^ nature of the posts. Compared to newspaper language which involves ample editing and scrutiny, or even recorded conversational data, in which speakers are aware of the fact that they are being recorded, Twitter affords a rapid and uncensored window into off-the-cuff language use.

A second observation to be made about the function of hashtags on Twitter is that, as argued in section 4.1, while it is true that hashtags can and do function as affiliative tags and categorizing and community-building devices at a macro-level (see discussion of the hashtag #banthehaka as a discoverable tag for joining the debate about the performance of the haka in rugby matches), they also have a purely semantic dimension, expressing actual linguistic content, at a micro-level. We hope to have shown that, while the two roles can sometimes fruitfully co-exist, there are also cases where one role is foregrounded to the partial or complete exclusion of the other. For instance, the semantic content of #honeyhui is more important than the categorizing function in example (20), and the affiliative role is primary for #banthehaka in example (8), rendering the semantic content of the hashtag obsolete.

### 5.3. Integratedness of Loanwords in Receiver Language

One final observation we make relates to what Twitter and hybrid hashtags might be able to tell us about loanword integration. The question of how to determine the entrenchment of loanwords within a receiver language is a longstanding problem (see discussion in Turpin, 1998; Jones, 2005; Zenner et al., 2014; Levendis and Calude, 2019). This issue is particularly problematic in the context of English as a receiver language because typical ways of establishing entrenchment of loanwords involve examining morphological and phonological integration of loanwords in the adoptive language, and English has a distinct lack of morphological marking^15^. Additionally, some studies cite listedness as a factor in establishing entrenchment (Stammers and Deuchar, 2012, p. 631), but recent work casts some doubt as to whether that is a robust measure for Māori loanwords in (New Zealand) English (Levendis and Calude, 2019).

Given the time and effort costs involved in obtaining the spoken language data required to tap into phonological integration, morphological integration remains a key factor in determining loanword entrenchment. As regards English, one of the few morphological strategies for signaling entrenchment of a loanword cited in the literature is plural marking (on nouns). However, for prescriptive reasons, this strategy has been actively discouraged in New Zealand with regard to Māori loanwords (see Davies and MacLagan, 2006, p. 90). Interestingly, there is one loanword which appears to be exempt from this “rule,” namely the loanword *kiwi* (*kiwis* does not appear to attract criticism)—this exemption is likely a sign of entrenchment in itself because it points to the fact that many speakers of New Zealand English are no longer conscious of the fact that *kiwi* is borrowed from Māori.

Our corpus of hybrid hashtags shows two further possible sources of evidence for loanword entrenchment, namely the use of loanwords in hybrid hashtags and the use of derivation. Because hybrid hashtags involve loanwords that have been found to be very frequent in other corpora (see discussion in section 4.1), it seems reasonable to assume that the presence of a hybrid hashtag involving a given loanword can be taken to be a sign of entrenchment of that loanword in English. Secondly, our corpus exhibits some (albeit few) examples of loanwords used with productive English derivational suffixes, see examples (23) and (24).

(23) I'm outnumbered in this café by French speakers. Rather cool. But it'd be better to only hear Te Reo. **#maorifynz** [ID 98119407166955520]

(24) Using te reo tongue-twisters makes even the simplest acting warm-up games tricky (and hilarious). **#maorifynz** [ID 169695510075158530]

Both the presence of derivation and the use of loanwords in hybrid hashtags are predictors of entrenchment; however, the absence of these is not necessarily an indicator of a lack of entrenchment.

## 6. Conclusion

This paper reports findings related to a set of productive hybrid hashtags, made up of lexical components from two separate languages, namely, a minority, indigenous language (te reo Māori) and a dominant lingua franca (English). The hybrid hashtags are extracted from a diachronic corpus of tweets, over a 10-year period between 2009 and 2018, and analyzed using a combination of descriptive and quantitative tools. The main contributions of this paper are as follows:

- described semantic and syntactic categories of hybrid hashtags, as well as their functions in discourse;
- proposed and operationalized a new metric for measuring the life-cycle of a hashtag, a hashtag's half-life;
- proposed additional criteria for measuring loanword morphological integration;
- studied the role of loanwords from te reo Māori in (primarily, New Zealand) English and society.

We find that Twitter constitutes a rich source of investigating loanwords and language-mixing phenomena, as well as informal, creative language use. The data analyzed show that hybrid hashtags are extremely versatile with regard to their length, semantic function and word-class, encompassing various types of each. Given that hybrid hashtags appear to be composed of loanwords which are known to be highly productive in other genres, we argue that the presence of a loanword in a hybrid hashtag could be a reliable predictor of loanword entrenchment.

Concerning hashtags more generally, the internal versatility of the hashtags we analyzed and the need for decomposition in order to decode their semantic content point to the fact that hashtags are best regarded as artificial words (and not true words), which cannot be derived through compounding or other traditional word-formation processes. Secondly, their function in discourse is of a dual nature: on the one hand, they have a micro-discourse role in which they carry semantic meaning (this can be downgraded or altogether canceled if it conflicts with their wider discourse function), and at the same time, they have a macro-discourse role in which they act as community-forming or categorizing devices (this can similarly be downgraded in favor of their micro-discourse role).

One cited benefit of analysing language on Twitter is the rapid nature of change, observable within a shorter time frame than linguists are typically used to Grieve et al. (2018), and hashtags, in particular, constitute a perfect example of a fast-changing, highly transient linguistic phenomenon. We problematize current measures of hashtag life-span, which take into consideration duration of existence, but neglect to measure overall impact, and propose a new measure of hashtag life-span, namely, the hashtag's *half-life*. We build statistical models which show that there are associations between linguistic properties of the hashtags analyzed and their half-lives, although these models currently suffer from several limitations (they are missing factors related to the content of the tweets containing the hashtags and features related to the user, such as gender and ethnicity)—limitations which we leave for future work.

## Data Availability Statement

The dataset generated for this study can be found on the Kiwi Words website at waikato.github.io/kiwiwords/hh_corpus.

## Author Contributions

All authors listed have made a substantial, direct and intellectual contribution to the work, and approved it for publication.

### Conflict of Interest

The authors declare that the research was conducted in the absence of any commercial or financial relationships that could be construed as a potential conflict of interest.

## Footnotes

^1^To the best of our knowledge, the examples of tweets we include in this paper comply with the terms and conditions specified by Twitter for research use, see https://developer.twitter.com/en/developer-terms/more-on-restricted-use-cases.html.

^2^In our data, we also included #hakarena, which comprises one morpheme (*-rena*, from *Macarena*) and one free word (*haka*).

^3^We use these terms interchangeably.

^4^The corpus is available to download at https://kiwiwords.cms.waikato.ac.nz/corpus/

^5^https://developer.twitter.com/en/docs/tweets/search/api-reference/get-search-tweets

^6^Waitangi Day is the national day of New Zealand, which takes place in February each year.

^7^Māori Language Week is an annual, government-sponsored initiative to promote Māori language use.

^8^The HH sub-corpus is available to download at https://waikato.github.io/kiwiwords/hh_corpus/

^9^We decided to include these factors because the hybrid hashtags in our dataset appear to show considerable variation in regard to both of these.

^10^All figures included are drawn using *R Software* (R Core Team, 2017) and the *ggplot* package (Wickham, 2009).

^11^We use number of years here rather than number of weeks or months for illustrative purposes, but the same argument holds for these measures.

^12^We first tried building a GLMM model with a Poisson distribution but this did not fit our data well (the overdispersion factor was 0.002004332), so we changed to a Quasi-Poisson distribution which performed much better (the overdispersion factor for the final minimal adequate model was 1.225681).

^13^We are grateful to Laurie Bauer for his input which shaped this proposal.

^14^Some people do not use their real names on Twitter.

^15^There is a wealth of work being done on phonological integration of loanwords, too large to cite here, but for a recent and meticulous study of phonological integration of Māori loanwords in New Zealand English, see Hashimoto, 2019 and references cited within.
