# Supplementary material for: Hybrid Hashtags: #YouKnowYoureAKiwiWhen Your Tweet Contains Māori and English
Source: Front Artif Intell. 2020 Apr 9;3:15. doi: 10.3389/frai.2020.00015 (PMC7861263; doi:10.3389/frai.2020.00015)
Supplement: Supplementary file 1 [file Data_Sheet_1.PDF]

Supplementary Material

1 SUPPLEMENTARY DATA

See https://waikato.github.io/kiwiwords/hh_corpus/.

2 SUPPLEMENTARY TABLES AND FIGURES

2.1 Data Collection

We extracted 287 hybrid hashtags from the MLT corpus. The hashtag #mycrazywhanau is hybrid, because it contains lexical items from both English ("my", "crazy") and Māori ("whanau"). A hashtag such as #KiaKahaTeReoMaori is non-hybrid, due to the absence of English. Similarly, the hashtag #nativeaffairs is non-hybrid, due to the absence of Māori.

We devised the following set of rules to merge hashtags with similar variants:

1. Convert all letters to lowercase. In this way, #GoKiwis and #GOKIWIS were merged with #gokiwis.
2. Remove any macrons that occur in the hashtag. For example, #beingmāori was merged with #beingmaori.
3. Remove plural forms (ending with -s). For example, #flyingkiwis was merged with #flyingkiwi.
4. Merge hashtags that differ by only a specific word (the indefinite article "a"), or suffix ("-in(g)"). Consequently, #proudtobeakiwi was merged with #proudtobekiwi, #youknowyoureakiwiwhen became #youknowyourekiwiwhen and #keepinitreo became #keepitreo.

2.2 Data Analysis

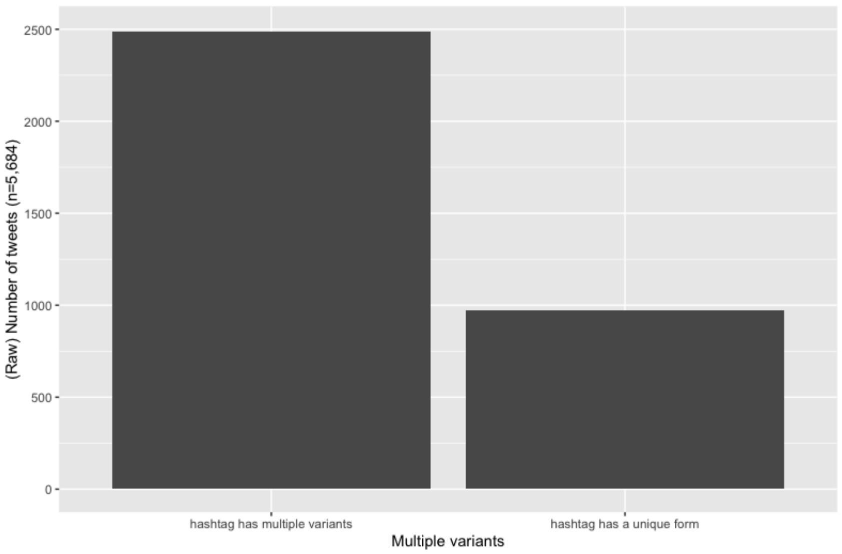

Figure S1: Distribution of hashtags with and without variants in the hybrid hashtag set.

The table below gives the Kendall Tau results for the 81 hybrid hashtags we analysed. The hashtags are given in order of overall frequency of use, from most to least frequent. There were 18 hashtags that had statistically significant Kendall Tau scores (given in bold).

Table S1: Kendall Tau results for the hybrid hashtags (calculated across months).

**Hybrid Hashtag**	**Kendall Tau Score**	**p-value**
**#WaitangiDay**	**0.271**	**3.08E-05**
#MaoriLanguageWeek	0.0856	0.19477
#GoKiwis	0.0714	0.28384
**#proudkiwis**	**0.193**	**0.0056741**
#kiwipride	-0.0784	0.26065
**#LetsShareGoodTeReoStories**	**0.237**	**0.00079501**
#proudtobeakiwi	-0.084	0.24356
#youknowyoureakiwiwhen	0.0827	0.25675
**#kiwifruit**	**0.158**	**0.030204**
#growingupkiwi	0.11	0.12293
**#Hakarena**	**0.186**	**0.010099**
#kiwiscanfly	-0.0691	0.3469
**#kiwiproud**	**0.164**	**0.025489**
#gothekiwis	-0.00834	0.9104
#UpTheKiwis	0.126	0.082197
#kiwisareawesomepeople	-0.0219	0.76893
#beingmaori	0.0659	0.38244
**#kiwilove**	**0.154**	**0.035347**
#KeepinItReo	0.127	0.086416
#TreatyofWaitangi	-0.0497	0.50243
#flyingkiwis	0.0912	0.21878
#kiwimusic	0.00926	0.90396
#NZMaori	0.0323	0.66459
**#kiwias**	**0.173**	**0.020416**
#kiwipower	0.0935	0.20978
#KiwiTreason	0.063	0.39914
#kiwifacts	0.101	0.17508
**#KiwiSongs**	**0.161**	**0.030353**
#ilovekiwis	0.0401	0.59232
#BanTheHaka	-0.0228	0.76107
**#maorilanguage**	**0.277**	**0.00018179**
**#HakaTime**	**0.201**	**0.0062338**
#hugakiwiday	0.0194	0.79633
#lovekiwis	0.0331	0.65747
#LetsGoKiwis	0.064	0.39016
#kiwilife	0.0547	0.46585
#KiwisAllDay	-0.0731	0.33091
#kiwisabroad	0.0243	0.74428
#MAORISTYLES	-0.0589	0.42652

**#thehaka**	**0.144**	**0.056284**
#kiwiproblems	0.086	0.24205
#kiwislang	-0.107	0.14948
#loyalkiwis	-0.0147	0.84625
#kiwibird	-0.037	0.62064
#kiwigirl	0.0393	0.5984
#KiwiQuestion	0.0472	0.52665
#kiwisrule	-0.00939	0.90268
#kiwiaccent	0.037	0.6236
**#maoripride**	**0.207**	**0.0055875**
**#MeanMaori**	**0.142**	**0.056923**
#huitweet	-0.0212	0.77826
#kiwifilms	-0.0528	0.48257
#KiwiSayings	0.0337	0.65237
#kiwisrock	-0.00921	0.90558
#maoriculture	0.0839	0.26461
**#kiaora4that**	**0.217**	**0.0037**
#kiwigold	0.0407	0.59068
#lovethehaka	-0.0078	0.92045
**#ProudMaori**	**0.15**	**0.045671**
#whatkiwisdo	0.134	0.072893
#AotearoaNZ	0.129	0.085903
#goodtereostories	0.0998	0.18477
#kiwilegend	-0.132	0.078489
#maorifynz	0.0491	0.51542
**#NewKiwiBurgerSong**	**-0.162**	**0.031091**
#kiwichicksrock	-0.0775	0.3028
#kiwikids	-0.00692	0.93465
#kaitime	-0.0541	0.47505
**#kiwiberries**	**0.185**	**0.013808**
#maoristudentsuccess	0.0746	0.32515
#kiwiasbro	0.00692	0.93465
#KiwisDoFly	0.0899	0.23448
#nokiwiemoji	-0.00999	0.90211
#honeyhui	0.105	0.16584
#kiwistyle	0.0683	0.37083
#proudtobemaori	0.0423	0.58335
#replacemoviequoteswithkiwi	0.0683	0.37083
#kiwiemoji	-0.0553	0.47047
#Moviequoteswithkiwi	0.0553	0.47047
#replacesongwordswithkiwi	-0.0618	0.41891
#honorarykiwi	-0.0618	0.41891

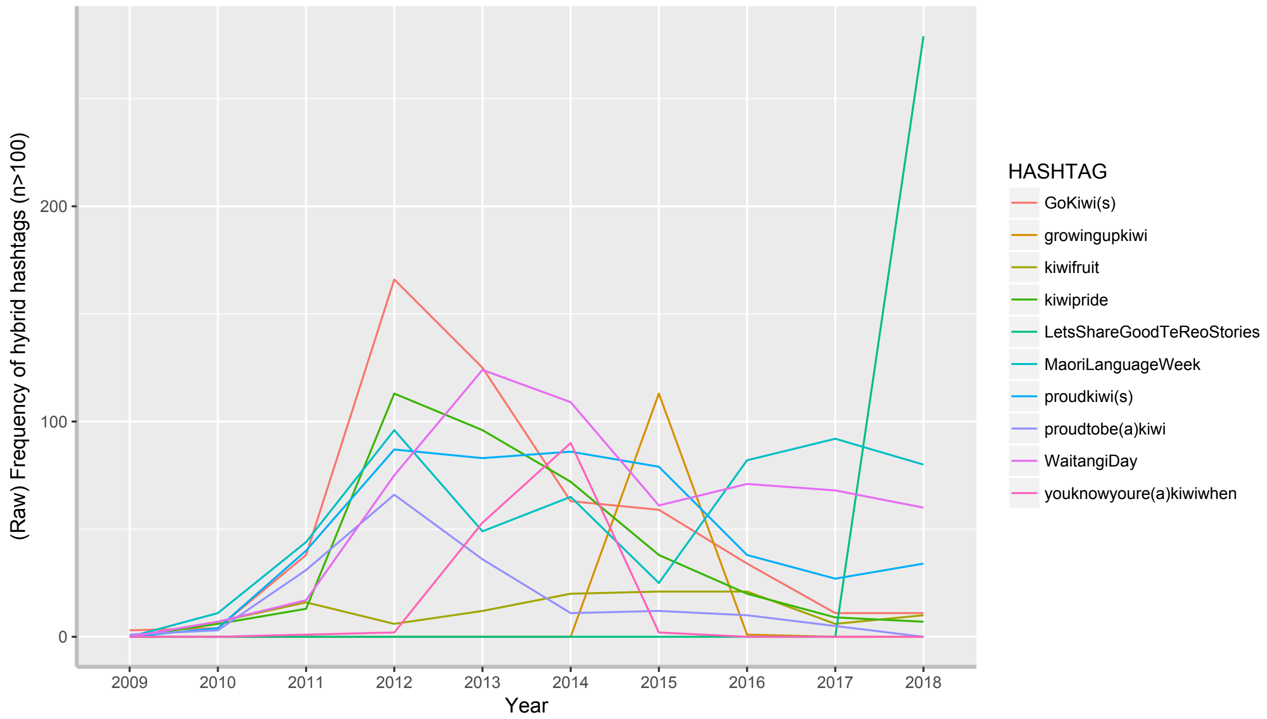

Figure S2: Diachronic plot of the ten most frequently occurring hybrid hashtags.

2.3 Modelling

Table S2. Variables included in the regression GLMM model.

Variable	Type	Meaning
hashtag	categorical	The hybrid hashtag used (preceded by the '#' symbol).
user_freq	numeric (discrete)	The total number of tweets in which that particular Tweeter uses that particular hashtag.
num_tweets	numeric (discrete)	The total number of tweets in which that particular hashtag occurs in the corpus (across all Tweeters who use it).
multiple_variants	binary	Some hashtags have multiple variants (e.g. #kiwiGold and #kiwigold). 0= hashtags with only one variant, 1= hashtags with multiple variants.
half_life (months)	numeric (discrete)	Integrate over the total existence of the hashtag and take 50% of that area, then calculate how long it takes for the hashtag (in number of months) to reach this half-life point, from the time of its first use (range: 0-79 months).
words	categorical	The number of words that are used to make the hashtag (range, 2-6).
wordclass	categorical	The syntactic word-class of the hashtag (nominal vs. non-nomimal).
semantic_domain	categorical	The semantic function (broadly speaking) of the hashtag (NZ identity, Māori culture, sport, and other).

Table S3. Summary of the final minimal adequate model. The bold text represents an interaction between the corresponding columns.

**Semantic Domain**	**Length**	**Word-Class**
Chisq=68329.13, df=3, p<2.2e-16	Chisq=722.56, df=1, p<2.2e-16	Chisq=14984.10, df=1, p<2.2e-16
Chisq=553.38, df=3, p<2.2e-16		
Chisq=102.50, df=3, p<2.2e-16		Chisq=102.50, df=3, p<2.2e-16
	Chisq=425.00, df=1, p<2.2e-16	
Chisq=56.77, df=3, p<2.878e-12		

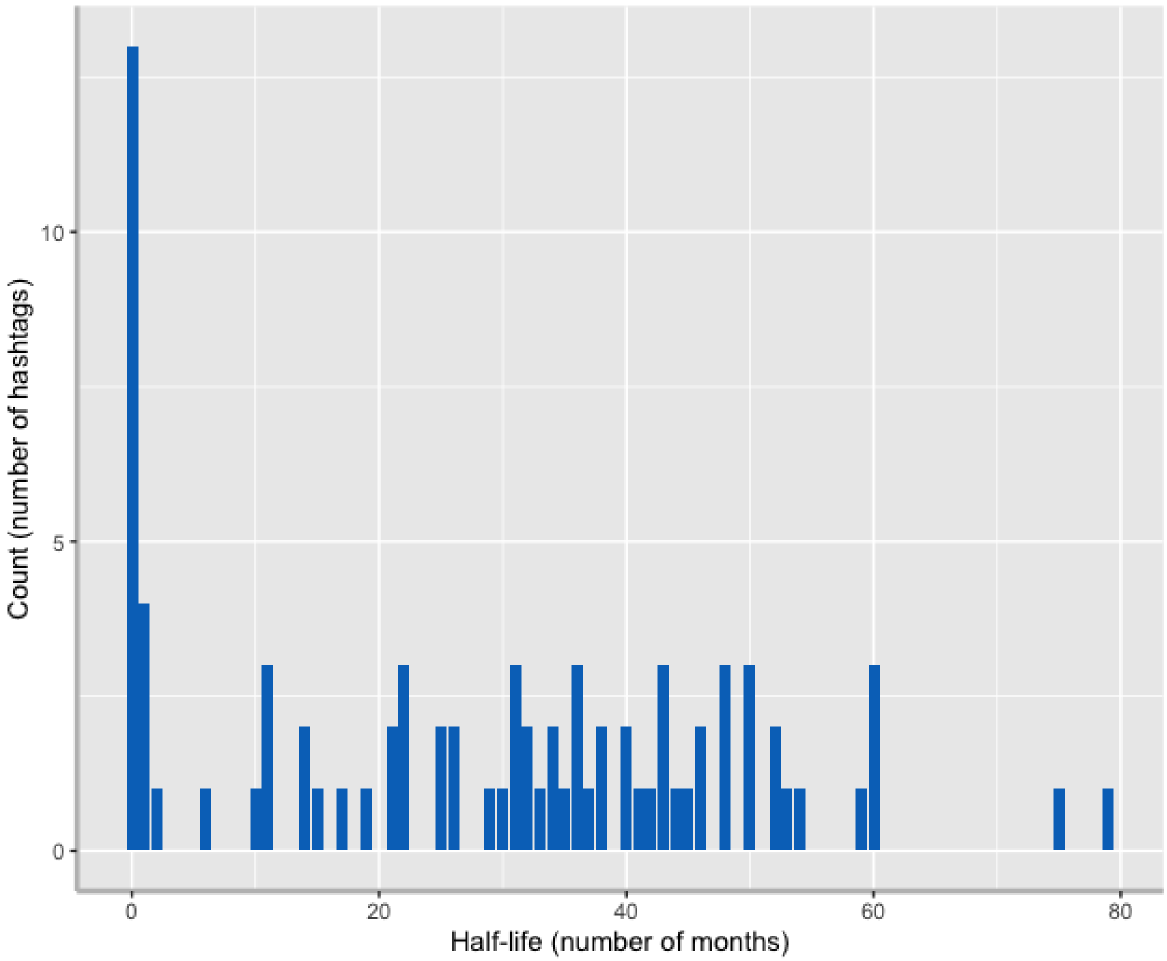

Figure S3: Frequency distribution of the various half-lives of the 81 hybrid hashtags.

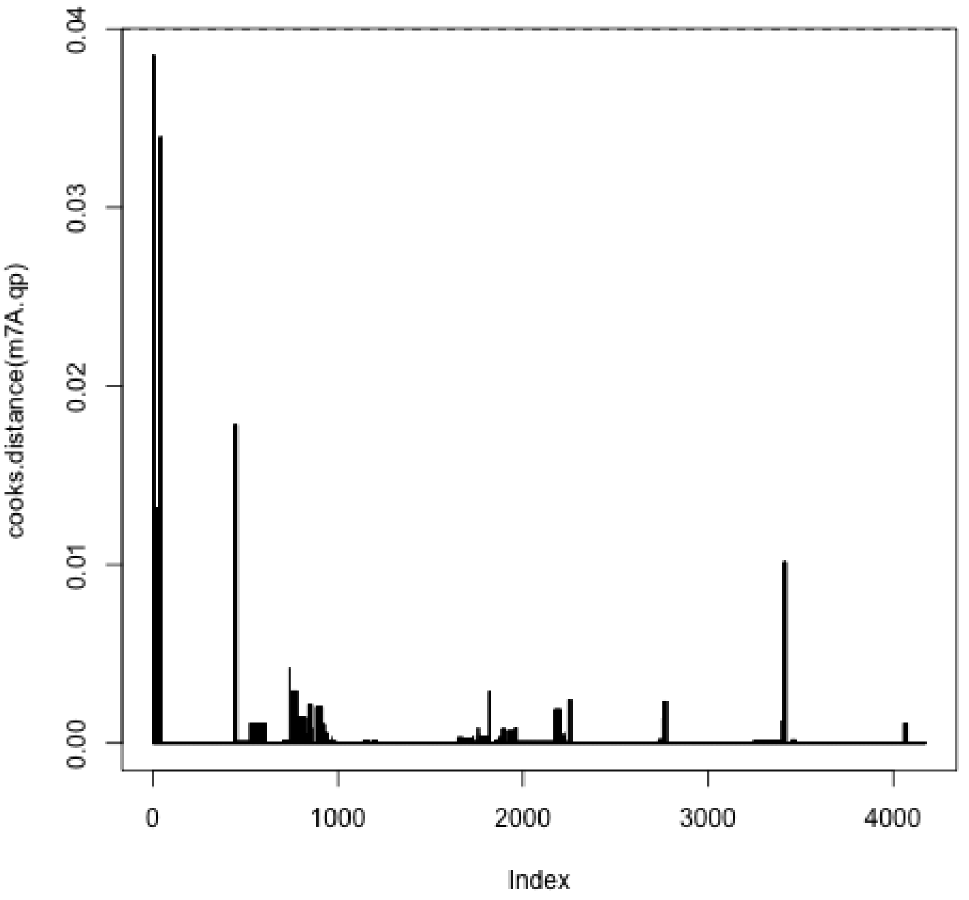

Figure S4: Cook's Distance Plot for the equivalent Fixed-Effects model.
